# Health and safety considerations for healthcare simulation: a scoping review of published literature

**DOI:** 10.1186/s41077-026-00443-w

**Published:** 2026-05-02

**Authors:** Angela O’Dea, Paul O’Connor, Sinéad Lydon, Bronwyn Reid-McDermott, Dara Byrne

**Affiliations:** 1 Irish Centre for Applied Patient Safety and Simulation, School of Medicine, University of Galway, Galway, Ireland; 2 Discipline of General Practice, School of Medicine, University of Galway, Galway, Ireland; 3 Health Service Executive, National Simulation Office, Dublin, Ireland

**Keywords:** Health and safety, Healthcare simulation, Risk management, Scoping review

## Abstract

**Background:**

The unique environments created within healthcare simulations may create distinct health and safety risks for patients, learners, simulation faculty and staff, and other participants involved in these activities. Guidance to aid simulationists to manage the physical risks arising from simulation activities is limited and there is no integrated synthesis of known risks or risk mitigation strategies.

**Objectives:**

To identify and examine literature addressing the physical health and safety risks associated with healthcare simulation, in order to inform the development of effective safety management strategies.

**Methods:**

This review included published empirical research and non-empirical literature (e.g., commentaries, editorials) examining physical health and safety risks associated with any form of healthcare simulation programme, event, or facility. A multi-pronged search strategy was used including electronic databases, Google Scholar searches, and reference list searches of literature published between 2010 and 2025. Risks, contributory factors and mitigation strategies were identified and mitigation strategies were coded according to the Hierarchy of Controls framework.

**Results:**

Sixteen articles were included. The literature most frequently identified physical health and safety risks to patients during in-situ simulation. Risks to participants and simulation staff or faculty were reported less often and primarily related to exposure to clinical equipment and musculoskeletal injury associated with the physical demands of simulation. Risks to simulated participants included clinical interventions being performed on them. Contributory factors included learner inexperience and failure to recognise risks, rule violations, pursuit of realism in simulation, poor simulation design, inadequate preparation, and lack of formal safety systems. Mitigation strategies were predominantly administrative, with rules, procedures, and checklists reported in 94% of sources, while elimination and engineering controls appeared in 37% and 19% respectively. There was call for clinical governance tools and processes to support a robust simulation health and safety approach.

**Conclusions:**

These findings highlight the need for co-designed, simulation-specific governance tools, including standardised risk assessments, adverse event reporting systems, and safety policies tailored to simulation environments. These tools should be embedded within, and aligned to the parent organisation’s health and safety governance process, rather than constituting a separate or parallel governance process.

**Supplementary Information:**

The online version contains supplementary material available at 10.1186/s41077-026-00443-w.

## Background

Healthcare simulation uses tools, environments, or scenarios that mimic clinical care so learners can practice safely before treating real patients. Common methods include task trainers (e.g., airway models), manikins in simulated settings (like operating rooms or Intensive Care Units (ICUs)), simulated patients, augmented reality (AR) or Virtual Reality (VR) technologies, and biological materials such as cadaveric or animal tissue. These approaches can be combined within a single simulation event. The goal of simulation is to improve healthcare providers’ skills and ultimately enhance patient safety and quality of care.

Patient safety is core to healthcare simulation. Yet simulation has the potential to cause both psychological and physical harm to patients, learners, simulation staff and faculty, and simulated participants (SPs). The psychological risks associated with simulation have received considerable attention in the simulation literature [1–3]. However, the physical risks of simulation have received much less attention, but are no less important [4]. This review focuses on the physical risks of healthcare simulation.

Simulation sits in a space which is neither a clinical environment, a laboratory environment nor an educational environment, but has characteristics of all three. This convergence of characteristics creates specific conditions that may increase the physical risks faced by simulation participants. First, simulation uses the tools and equipment of the clinical environment but the tools are sometimes used in unusual or non-standard ways, and in an unintended context. It is common for equipment that no longer meets operational standards to be donated to a simulation programme. As such, it may not be subject to the same level of inspections and maintenance that are mandated in the clinical environment. In such situations equipment can function in ways that are not anticipated [5, 6].

Second, simulation sometimes involves people and systems being engaged in unusual ways and for purposes that are outside of normal scope of practice. For example, healthcare staff may train for situations that are unusual such as a fire in an operating theatre or an emergency transport in an ambulance. Moreover, simulation often involves individuals and teams who may not be familiar with each other, undertaking tasks that may be at the edge of their skills and capacity, in an unfamiliar environment. In the safety science literature, non-normal operations i.e. those that deviate from standard or expected conditions, are regarded as higher risk than normal operations as they involve uncertainty, time pressure, and reduced system predictability, all of which increase the likelihood of human error and system failure [7].

Third, simulation shares certain characteristics with a laboratory, particularly in its use of biological or chemical agents, and its focus on training high stakes procedures [8]. Laboratories are highly proceduralised spaces where activities are governed by strict protocols, regulatory oversight, and routine inspections [9]. By contrast, simulation centres often operate in a more flexible, ad-hoc way with less emphasis on the safety infrastructure [10]. In addition simulation environments tend to be far less regulated, often relying on locally developed, safety policies [4, 11] leading to increased potential risks.

Finally, simulation differs from other educational environments which tend to be low risk and theory based. In contrast simulation encourages learners to ‘have a go’, and to practise using equipment, tools, and techniques that they may not have mastered yet [12]. This pedagogical approach supports experiential learning but can also introduce safety risks. Participants in simulation are often inexperienced and still developing the knowledge and skills required to handle complex clinical equipment safely [13]. These factors combine to make the simulation environment inherently riskier than a traditional academic setting [14].

Thus, the unique environment of simulation may create substantial risks to the physical wellbeing of patients, staff, learners, and other participants involved in simulation activities [15]. These risks are not limited to those directly participating in the simulation; they may also extend to individuals in the surrounding clinical environment - including patients. Moreover, if simulation spaces and materials are not appropriately managed, hazards may persist beyond the duration of the simulation event itself. Simulationists have a clear duty of care to all individuals involved in, or potentially affected by, simulation activities. Yet there is limited literature on the physical risks of simulation [15], suggesting that these risks are underappreciated by the simulation community.

A recent review study by Lambert et al. [15] mapped the literature on risk assessment in health education simulation in order to identify how risk is assessed, measured and mitigated in that context. They found a dearth of literature on proactive risk assessment and called for a structured approach to risk assessment in simulation. Our study expands on the scope of the Lambert et al. study by moving beyond risk assessment in order to provide a synthesis of the literature considering the physical health and safety risks and risk management practices associated with all simulation contexts, including in-situ simulation. The findings will support the development of health and safety management strategies and best practice guidance for simulation, ensuring that simulation not only *teaches* safety but also *embodies* it. The research questions are:

- What are the reported physical health and safety risks of healthcare simulation and who is at risk?
- What are the reported contributory factors to healthcare simulation risk?
- What are the suggested mitigation strategies, to manage simulation risks?
- How do the suggested mitigation strategies map to the Hierarchy of Controls framework?

## Methods

The scoping review was conducted following the five-stage methodological framework proposed by Arskey and O’Malley in 2005 [16]. The review is reported according to the Preferred Reporting Items for Systematic Reviews and Meta-Analysis extension for Scoping Reviews checklist (PRISMA-ScR) [17]. As the included evidence was expected to be conceptually and methodologically heterogeneous, we integrated a narrative synthesis approach guided by the framework described by Popay et al. [18]. The purpose of the synthesis was to systematically organise and interpret findings, explore relationships within and between studies, and develop an overarching conceptual account of the literature. A review protocol was developed and agreed a priori within the research team but this was not made publicly available.

### Stage 1: identify the research question(s)

The purpose of this scoping review was to identify and examine the literature that considered the physical health and safety risks associated with any form of healthcare simulation; therefore, this review aimed to address a four-fold research question exploring what the risks of simulation are and who is at risk, what factors contribute to these risks, which mitigation strategies are used to manage them, and how robust the mitigation measures are. Search tactics, inclusion criteria and synthesis methods were explicitly aligned with this focus.

### Stage 2: identify relevant articles

Preliminary, exploratory searches of MEDLINE and Google Scholar were undertaken to identify relevant articles. The text within titles and abstracts, and the keywords used within these were used to support the subsequent development of a full search strategy for MEDLINE, CINAHL, and Web of Science that comprised of subject headings and free-text keywords (online supplemental Appendix 1 presents the MEDLINE search strategy and this was adapted as necessary for the other databases). Searches were undertaken in June 2025. Google Scholar was searched using combinations of simple search terms (e.g., “health and safety” AND “healthcare simulation”, “clinical simulation” AND “physical safety”, “risk management” and “healthcare simulation”). The first 100 returns from each search were screened for relevant articles not captured by the database searches. Finally, the reference lists of all included articles were screened to identify additional potentially relevant articles.

#### Study eligibility

Articles published between January 2010 and June 2025 were included in this scoping review. The review commenced in 2010 to reflect significant advances and expansion in healthcare simulation, which increased the scale, complexity, and associated physical risks of simulation-based education [19, 20]. Foreign language articles were excluded because of the cost and time involved in translating material. It is recognised that potentially relevant material may have been missed.

Articles were included if: they identified or considered simulation risks; explored contributory factors and/or discussed or described risk management strategies; they considered risks to patients, staff, learners, simulated participants (SPs), faculty or any other simulation participants, within or around healthcare simulation facilities; were written in English. All peer-reviewed publication types including research studies, review studies, commentaries and editorials were included. Inclusion of non-research literature is appropriate when peer-reviewed studies alone would offer an incomplete representation of current knowledge [16, 21]. Articles were excluded if: they were concerned with health and safety not specifically related to simulation activities (e.g. defibrillation safety during actual clinical practice); they only addressed psychological risk and safety; were concerned with health and safety risks specifically related to the COVID-19 pandemic.

### Stage 3: article selection

Titles and abstracts of all articles identified during the electronic searches were screened by two authors (AOD and POC) between July 2025 and August 2025. The full texts of articles that appeared eligible for inclusion, or articles in which the title and abstract did not provide sufficient information for the determination to be made, were sourced. AOD and POC reviewed all full-text articles. Decisions on eligibility were made through discussion and consensus. Reasons for exclusion at this full-text stage screening stage were documented by the authors.

### Stage 4: chart the data

**Fig. 1:**
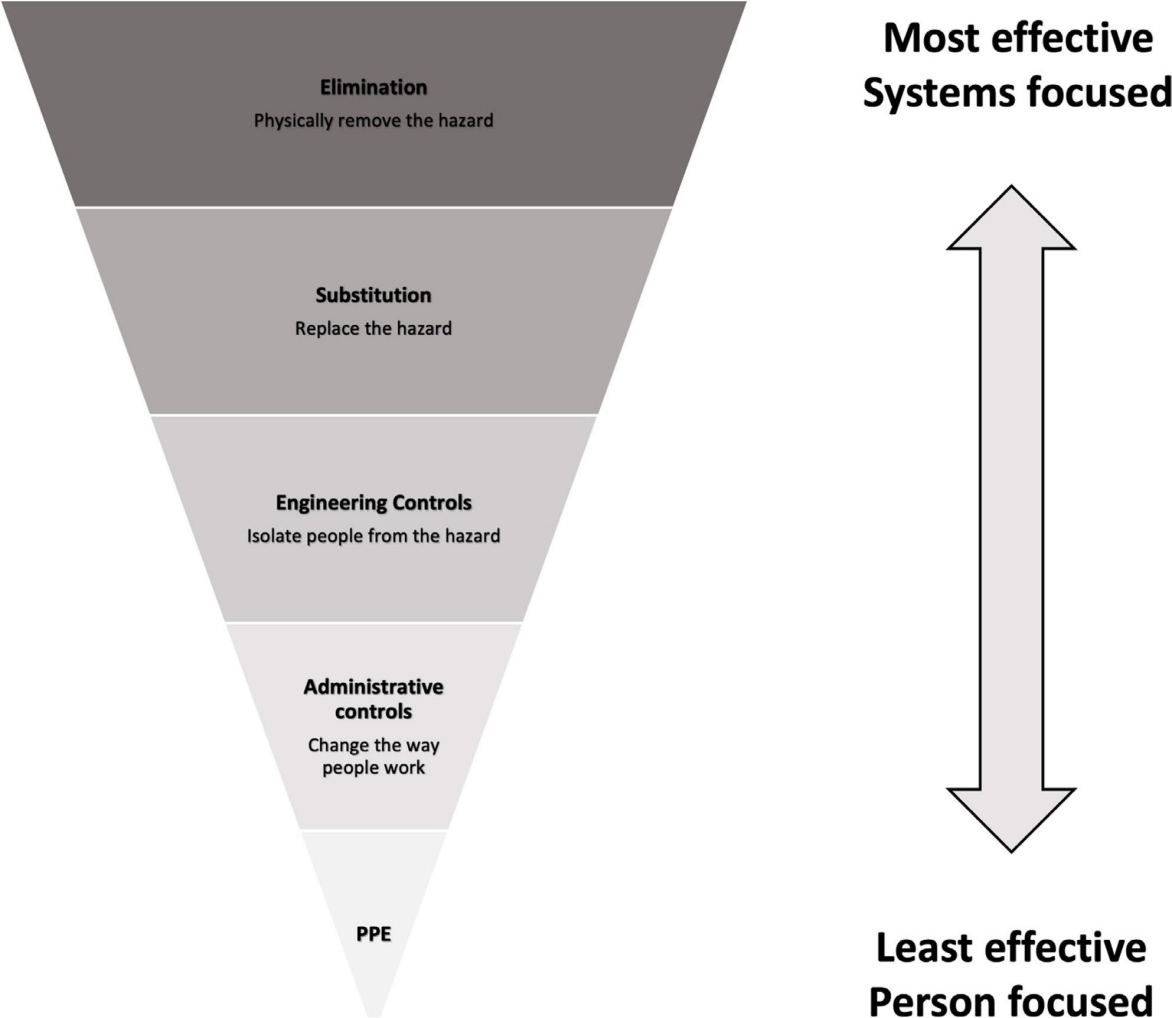
Hierarchy of Controls framework of risk control measures [22]

A two phase process was used to chart the data. In phase one, a data charting form was developed in accordance with best practice [21] and piloted by the reviewers with discussion and adaptation of the form and process as necessary. Ultimately, the reviewers extracted data on: article title, author(s) and year of publication, the country the study/article was conducted in/authored from, the type of publication (i.e. commentary, research article, editorial etc.), the article’s focus and aims, the risks identified, the contributory factors proposed, the mitigation strategies recommended (See Data Extraction Table in the online supplemental materials). Data were extracted by AOD and POC independently and a final data extraction record was agreed through review and discussion. In phase two, any mitigation strategies reported within studies were coded according to the Centre for Disease Control (CDC) Hierarchy of Controls framework (HOC) [22]. The HOC is a structured system for reducing or eliminating hazards by prioritising the most effective risk-control measures. It ranks controls from strongest to weakest. Mapping the mitigation strategies to the HOC allow us to assess whether articles report use of higher-order controls (such as elimination or substitution), which are inherently more reliable, or lower-order controls (such as administrative measures or personal protective equipment), which depend more heavily on human behaviour. This ensures that the analysis does not treat all interventions as equally effective when, in practice, they are not. Figure 1 presents the HOC framework, adapted from the Centre for Disease Control (CDC) [22].

### Stage 5: collate summarise and report the results

Following best practice guidance [18], synthesis occurred in three stages. In stage one a textual summary of each included article was developed and initial thematic categories were developed inductively. In stage two variations in setting, persons at risk, and primary risk focus were examined for their contribution to differences in causal factors and mitigation strategies. Interpretations were refined iteratively. Where constructs were used inconsistently across studies, interpretative translation was undertaken to clarify conceptual meaning. In stage three, findings were integrated into narrative accounts that addresses the research questions. The synthesis emphasised explanation and interpretation rather than quantification consistent with scoping review methodology.

## Results

**Fig. 2:**
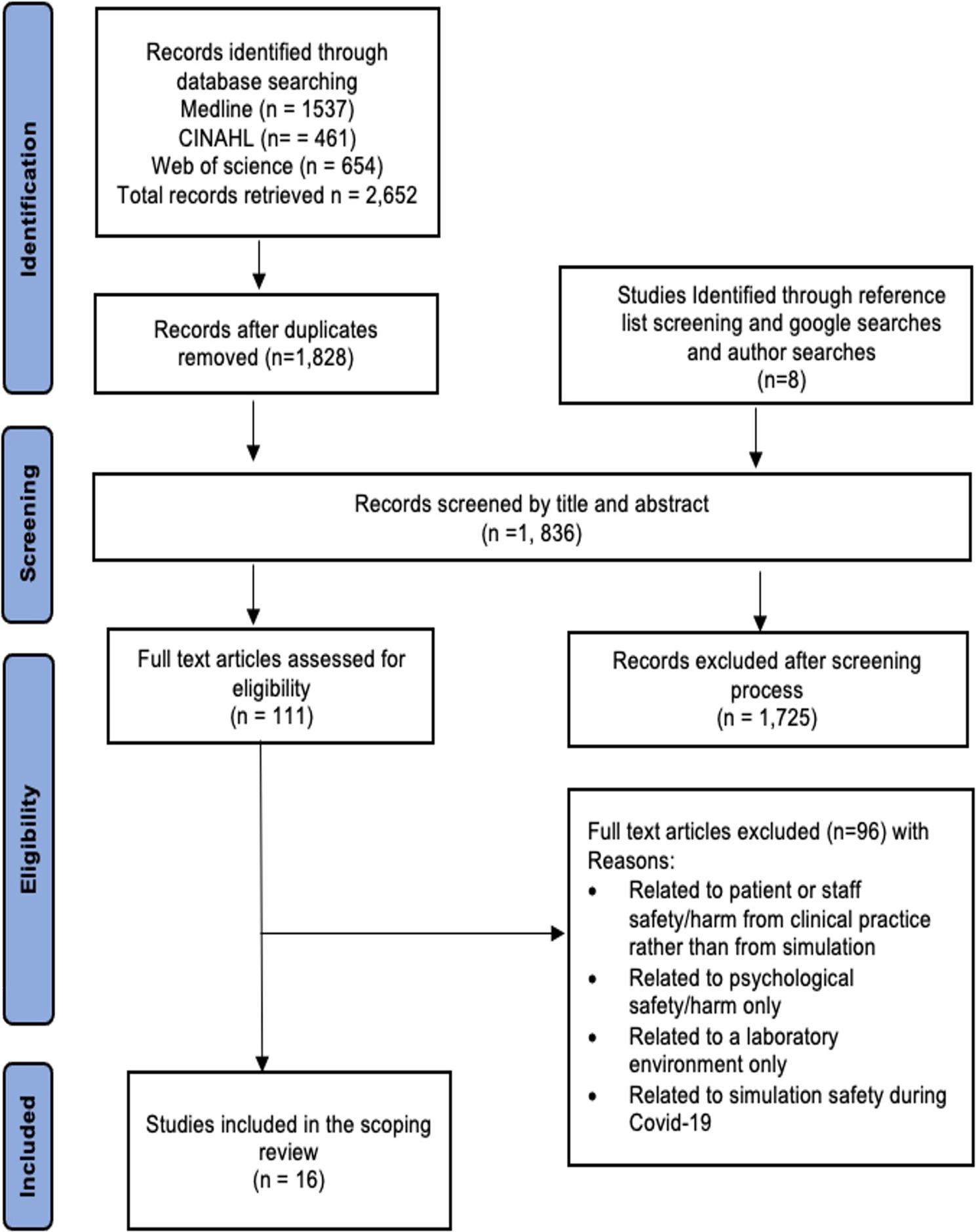
PRISMA Flow diagram depicting the processes of searching, screening, and selection

A total of 1,836 articles were identified from electronic database searches (see Fig. 2). Ultimately, 16 articles were identified for inclusion following the completion of searches and reference list screening. A detailed, study-by-study, data extraction record is presented in the online supplemental materials.

### Included studies

An overview of the characteristics of included articles is shown in Table 1. Four empirical studies were identified. Of the included articles, seven articles discuss risks that are location generic, that is, the risks can emerge in either a simulation centre or in-situ simulation such as defibrillation risk or manual handling risk, five articles discuss in-situ simulation risks and four studies relate to simulation centre risks (see Table 1. Article characteristics).

**Table 1:** Article characteristics

Descriptor	Studies, *n* (%)
Publication year	
2010–2014	3 (19%)
2015–2019	9 (56%)
2020–2025	4 (25%)
Country of origin	
USA/Canada	9 (56%)
Australia/New Zealand	4 (25%)
UK	2 (12%)
Publication type	
Commentary, Guidance, Special feature, editorial, viewpoint	8 (50%)
Research article/audit	5 (31%)
Review article	1 (6%)
Standards document	1 (6%)
Book chapter	1 (6%)
Simulation setting	
Location generic	7 (44%)
In-situ simulation	5 (31%)
Centre/facility based	4 (25%)
Person(s) at risk	
Patients only	5 (31%)
Patients and learners	3 (19%)
Participants (Learners and staff)	5 (31%)
Staff only	1 (6%)
SPs	2 (12%)

### What are the reported physical health and safety risks of healthcare simulation and who is at risk?

The risks most frequently identified in the literature were the risks to patients arising from in-situ simulation. Chief amongst these was the risk of contamination of the real clinical environment with simulation-only medications, equipment, or supplies [11, 23–27]. A second major risk involves compromising patient care by diverting essential resources such as staff, equipment, personal protective equipment (PPE), or institutional systems like first responders, for simulation activities ([4, 11, 23],– [26]). Negative learning, was also identified as a risk to patients as it can result in the transfer of incorrect knowledge, skills or behaviours into clinical practice [11, 26, 28]. Risks to participants and simulation staff and faculty were discussed less frequently and mainly related to exposure to clinical equipment such as defibrillators, sharps, electricity, fluids, and gases [5, 15, 28–31]; and musculoskeletal injury resulting from the physical demands of simulation work [15, 32]. For SPs there was a recognised risk that SPs may have interventions performed on them, if the learning environment is not carefully controlled [33, 34] (see Table 2).

**Table 2:** Primary risk focus and contributory factors

Primary risk focus	studies	%
Contamination of the clinical environment	7	(44%)
Misuse of resources/ interference with actual patient care	6	(38%)
Use of clinical equipment, electrical devices, fluids, gasses	4	(25%)
Negative learning e.g. no learning occurs or failure of learning to transfer to clinical practice	3	(19%)
Sharps	3	(19%)
Defibrillation safety	3	(19%)
Musculoskeletal injury	3	(19%)
SP safety	2	(12%)
Fainting, falls and other medical episodes	2	(12%)
Spreading infection	1	(6%)
Learners using unsterile supplies to practice on each other	1	(6%)

Contributory factors	studies	%
Learner lack of experience, not following rules, and lack of attention to personal safety	4	25%
Pursuit of hyper realism: Repurposing of clinical equipment for the simulation environment	4	25%
Lack of systems, approaches, processes, and policies for managing risk	3	19%
Poor simulation design, poor timing/planning, lack of learner education	3	19%
Under-appreciation of risks by simulation staff	2	12%

### What are the reported contributory factors to healthcare simulation risk?

Several contributors to risk in simulation were proposed in the literature. Learner-related factors such as inexperience, not following rules, and limited attention to personal safety were highlighted in multiple papers [5, 26, 29, 30]. The drive for realism through the use of modified or repurposed clinical equipment, was also identified as a risk factor [5, 11, 25, 31]. A lack of formal safety systems or policies was noted in several papers [15, 23, 32]. Other authors identified poor simulation design and insufficient learner preparation as contributory factors [23, 25, 28]. Finally, some papers reported that staff may fail to recognise simulation-specific risks [24, 26]; (see Table 2).

### What are the suggested mitigation strategies, and how do they map to the HOC framework?

Table 3. shows that administrative or training strategies were the most commonly reported mitigation approaches, identified in 15 of 16 sources (94%). Rules, procedures, policy and checklists were most common [5, 11, 23, 27, 29], followed by training and supervision [11, 26, 30–32]. Labelling, tracking and controlling strategies were commonly reported [5, 25–27]. Good instructional design including planning, briefing, supervision, monitoring, rotation, schedules, breaks were the strategies used protect SP wellbeing [33, 34]. The adoption of a formal ‘safety pledge’ [11] and clarification of job descriptions for simulation technicians [32] are other administration strategies.

**Table 3:** Mitigation strategies mapped to HOC framework

Level 1: Elimination strategies	8/16	50%
— ‘No go’ policy	4	25%
— Using only real medications for in-situ simulation	3	19%
— Instructional design to eliminate risk to SPs	2	12%

Level 2: Substitution strategies	0	0
Level 3: Engineering Strategies	3/16	50%
— Equipment purchasing, safety devices, inspections and maintenance	2	12%
— Facility design	1	6%

Level 4: Administrative strategies	15/16	81%
— Rules, procedures, processes, regulations, checklists, compliance	13	81%
— Training, supervision, preparation of learners, prebriefing, education	8	50%
— Labelling,	4	25%
— Accounting, tracking	4	25%
— Separate systems and controlling access	3	19%
— Accurate job descriptions	1	6%
— Adopt the (FHSS), 10-item “pledge”	2	12%
Level 5: PPE	1	6%

Elimination strategies such as ‘no-go policies’ were reported in 6 sources (37%). A ‘no-go policy’ states a set of conditions under which simulation will not proceed. For example, conditions of high patient acuity or short staffing may trigger a ‘no-go’ decision. The conditions are set in advance. Engineering strategies are recommended in in 3 of 16 sources (19%). Engineering controls include workspace and facility design [28, 32]; use of lifting devises to protect against musculoskeletal injury [32]; and equipment inspection and maintenance to reduce emergent equipment related risks [5]. Personal protective equipment (PPE) was rarely mentioned, appearing in only one source [5] (6%). Substitution strategies were not reported in any source (using only real medication could be considered a substitution strategy, but here it was coded as an elimination strategy since fake medications are eliminated from the environment). Overall, the findings indicate a strong reliance on administrative controls, with comparatively less emphasis on higher-order controls such as elimination and engineering, and minimal use of PPE. Table 3 shows the mitigation strategies mapped to the HOC framework.

Beyond the specific actions required to address individual risks, the literature highlights the need for governance strategies that manage health and safety more broadly. Three key health and safety governance strategies were identified in the literature: the development of a simulation safety policy ([4, 11, 23]); the proactive identification and assessment of risks [15, 30]; and strategies to report and learn from adverse events [5, 15, 23, 28]. Such tools will support greater awareness of the physical risks specific to simulation and the identification of appropriate mitigation strategies. Table 4. Presents each study charted on the inclusion criteria.

**Table 4:** Summary table of studies

Author and date	Risks identified and persons at risk	Contributory Factors proposed	Suggested mitigation strategies	Hierarchy of controls
Bajaj, K., et al. (2018). [24]	In-situ simulation: Risks to **patients** from: Simulated medications, soiled equipment, misuse of resources	Staff not recognising the risks of in situ simulation	Collaboratively develop and agree ‘no go’ rules	Elimination
Boyle, M. J., et al. (2015). [29]	Location generic risks: Defibrillation safety: The risk to simulation **participants** (including learners, staff, faculty) of being inadvertently shocked	• Lack of training and experience using the equipment • Students failing to follow procedures	• Rules for defibrillator safety • Training in defibrillation before use in training	Administrative/ training
Bradley, M. (2022). [33]	Location generic risks: The risk of physical harm to **SPs** from learners performing interventions on them	None identified	A SP safety checklist to be completed before and after the simulation event	Elimination Administrative
Brazil, V. et al. (2022). [4]	In-situ simulation: Risks to **patients** and health systems from: Simulated medications, misuse of: staff, emergency response systems, patient record systems, PPE, risk of spreading infection, injury from moving manikins	The close physical proximity of simulated and real practice	A simulation safety policy	Administrative
Hambridge et al. (2022). [30]	Simulation centre: Risks to **Learners** and **staff** from: Sharps, fainting, back injury, facial injury, slips, splash to the eye	• Lack of experience • Underdeveloped skills • Lack of attention to personal safety	• Use simulation to demonstrate best practice • Supervision of practice • Risk assessment • Policy for sharps disposal • Compliance with regulation • biosafety awareness	Administrative/ training
Hensel, D., et al. (2019). [32]	Simulation centre: Risk to **staff** from: Musculoskeletal disorders	Lack of systems to protect workers from harm	• Accurate job descriptions for simulation technicians • Written policies and procedures • Reporting systems • Engineering controls • Administrative controls • Individual level strategies e.g. instruction	Administrative Engineering
Lambert et al. (l2025). [15]	Simulation centre: Risk to **learners** and **staff** from: Musculoskeletal injuries, fainting and other medical episodes, needle stick /sharps injuries	Lack of a structured approach to risk assessment	• A structured approach to risk assessment • Simulation specific adverse event reporting	Administrative
Lewis, K.L.et al. (2017). [34]	Location generic risks: Risk to **SP** educators from: Breaches of safety, confidentiality and respect	None identified	13 practice guidelines to protect SPs Including: • Ensure safe working conditions e.g. rotations and breaks • Anticipation of hazards such as substances, sharps, defibrillators • Screening and opt out • Briefings, monitoring, reporting	Elimination Administrative
Marshall, S., & McIntosh, C. (2017). [28]	Location generic risks: Risks to **patients** and learners and staff from: Learning related adverse events Physical adverse events: electrical devices, defibrillators, clinical equipment, fluids, gas supplies, mannequins and sharps	• Poor simulation design • Poor planning and preparation for simulation.	• Good instructional design to anticipate and avoid risk • The design of simulation facilities • Good health and safety processes • Preparation of the learners • Strategies to learn from adverse events	Elimination Engineering Administrative
Morse, C., et al. (2019). [23]	Location generic risks: Risks to **patients** from: The accidental activation of resuscitation and emergency teams, Contamination of the clinical setting with simulation only equipment	Lack of: planning, policies and learner education	• Policies to prevent contamination • Prebriefing • Rules for learners and faculty • Reporting and investigation of adverse events	Administrative
Petrosoniak, A., et al. (2017). [25]	In-situ simulation: Risks to **patients** from: Interference with patient care, contamination of the clinical environment with simulation only equipment and medications	• Poorly timed simulations, • Equipment and roles different from the normal workplace	• Appropriate timing • A priori cancellation criteria • Labelling • Planning for equipment use and restocking • Checklists	Eliminating Administration
Raemer, D. B. (2014). [26]	In-situ simulation: Risks to **patients** from: Contamination of clinical environment with simulation only equipment, supplies and medications; Misuse of clinical resources Negative learning	Under appreciation of risks by staff, faculty and learners	• Develop ‘no go’ rules • Using real medications • Labelling • Controlling access • Accounting of medication, equipment and supplies • Education on the hazards of simulation • Policies and procedures	Elimination Administration
Raemer, D., Hannenberg, A., & Mullen, A. (2018). [11]	In-situ simulation: Risks to **patients** and **learners** from: Contamination of the clinical environment with simulation only equipment Learners using unsterile educational supplies Misuse of clinical equipment and institutional systems and resources including staff Negative learning	Modification made to suit simulation	• Cancelation protocol • Using real medications and supplies • Controlling access • Risk assessment • Adopt the (FHSS), 10-item “pledge” • Labelling • Accounting of medications and supplies. • Policies and procedures • Education on hazards of simulation	Elimination Administrative
Riem, N., et al. (2011). [5]	Location generic risks: Risks to **learners** and **staff** from: Real clinical devices and equipment being used in uncontrolled setting, risk from sharps, electrical equipment, fire, trip and falls	• The drive for realism in the use of medical equipment in simulation • Inexperienced trainees	• Identify hazards • Regular Inspections and maintenance of equipment • Strategy to ensure use of PPE • Incident reporting and analysis systems • Co design safety guidelines and a safety plan • Regulation of storage, use, and disposal of sharps • Compliance to standards and regulations	Engineering PPE Administration
Torrie, J., et al. (2016). [27]	Location generic: Risk to **patients** from: Contamination of the clinical environment with fake or expired medications intended for simulation use only	None identified	• Use only real, ‘in-date’ medications • Labelling • Separate systems for purchasing, handling and storage supply of fake medications • Tracking supplies and disposal • Manage all medications, fake or real, as required for real medications • Systems for pre-reconciliation and post-reconciliation • Rules for exit checking learners and staff	Administration
Turban, J. W., et al. (2010). " [31]	Simulation centre: The risks to **learners** and **staff** from: Poor defibrillation technique	The drive towards high fidelity simulation	• Defibrillator safety training for all users before defibrillator use • A safety policy, assuring user demonstration of minimum competency before use of live defibrillation during simulation training	Administration

## Discussion

This review sought to identify the key risks associated with healthcare simulation and to identify the contributory factors and strategies recommended to mitigate them. Only 16 studies were identified that directly address the issue of the physical health and safety risks of simulation. This is in stark contrast to the large volume of literature exploring the issue of psychological safety risks of simulation [4], and suggests that the physical safety risks of simulation are under-appreciated in the simulation literature.

### Simulation risks and who is at risk

The health and safety literature on healthcare simulation focuses primarily on risks to patients during in-situ simulation, reflecting the idea of patient safety as the primary driver for simulation in healthcare. Patient related risks (such as the diversion of clinical resources away from patient care and the contamination of the clinical environment with simulation-specific medications or equipment), are largely predictable and foreseeable. As such they can be mitigated through the establishment of organisational controls [24]. In contrast, risks to learners, simulation staff and faculty and simulated participants (SPs) receive comparatively limited attention within the health and safety literature. SPs are particularly vulnerable to harm due to their close physical proximity to dynamic learner behaviours and complex equipment and invasive procedures [33, 34]. SPs typically occupy a non-clinical role and may have limited familiarity with clinical protocols and standardised practices; this places them in a position of high exposure and limited control, a configuration known to increase accident likelihood [35]. Risks to learners, faculty and staff arise from the physically demanding, dynamic, and emergent nature of simulation work [32]. The pursuit of realism in simulation amplifies these risks by encouraging the use of complex clinical equipment in contexts for which it was not originally designed, (e.g., modified oxygen delivery systems or repurposed infusion pumps) [5, 11]. These risks reflect the properties of complex sociotechnical systems in which harm emerges from non-linear interactions between people, technologies, tasks, and environments rather than from discrete component failures [36]. Such risks are harder to predict and more challenging to mitigate.

### Contributory factors

A range of contributory factors to risk in healthcare simulation have been identified. At the individual level, a recurring theme is the under-appreciation of risk among staff, faculty, and learners, particularly in relation to in-situ simulation [24, 26]. This concern is supported by recent survey research, which found that it was common for simulationists to have limited or no training in simulation design or delivery [37–39]. Moreover, the safety science literature emphasises that hazards are more likely to result in harm when they are insufficiently anticipated and underestimated [40, 41]. Procedural non-adherence, including learners’ failure to follow established protocols and attend to personal safety, represents an additional concern [29]. In the safety science literature inexperience and lack of training and supervision can be considered latent conditions which can interact with active failures such as procedural non-adherence, to increase the likelihood of adverse events [42]. At an organisational level, established health and safety governance processes of the parent organisation, are not always effectively applied to simulation activities, undermining safe simulation delivery [16, 23, 32]. Risks are also exacerbated by poor simulation scenario design, inadequate preparation, poorly timed activities, and discrepancies between simulated and real-world equipment, roles, or workflows [25, 28]. Finally, the pursuit of realism, particularly through the modification or use of real medical equipment, may introduce avoidable hazards, especially when combined with learner inexperience [5]. These factors support a systems-based interpretation of risk in healthcare simulation, reinforcing the safety science assertion that harm is rarely attributable to individual behaviour alone but instead emerges from interactions between people, tasks, technologies, and organisational contexts [43].

### Mitigation strategies mapped to hierarchy of controls

Mitigation strategies can be considered in two broad categories: those primarily aimed at protecting patients during in-situ simulation, and those designed to safeguard other participants, including learners, faculty, staff, and simulated participants (SPs).

#### Strategies to protect patients during in-situ simulation

Reflecting the emphasis on risks to patients, the mitigation strategies focus primarily on preventing inadvertent harm to patients arising from in-situ simulation, through proactive risk management and organisational safeguards. The establishment of ‘no-go’ rules [24] exemplifies this approach. This is a particularly robust strategy from a safety science perspective, as it removes reliance on individual staff members’ judgement at the point of delivery and instead embeds decision-making within agreed organisational rules. Similarly, the use of real, in-date medications during in-situ simulation constitutes a reliable strategy for preventing contamination of the clinical environment [27]. Where elimination of risk is not feasible, administrative strategies such as procedures for tracking, handling, storing, and counting medications, provide additional protection [4]. Comparable strategies were recommended to mitigate the risk of contamination with simulation-only equipment [27]. Collectively, these approaches indicate that the simulation community has invested significant effort in developing systematic safeguards against risks to patients. When considered in relation to the HOC framework [22], patient-focused mitigation strategies are predominantly aligned with higher-order controls, particularly elimination [24, 25]. Where hazards cannot be fully eliminated, reliance shifts to administrative controls, which, are more vulnerable to human variability and system pressures.

#### Strategies to protect learners, faculty, staff and simulated participants

Strategies to protect participants such learners, faculty, staff, and SPs. are predominantly situated at administrative level. Rules, procedures, and checklist are the most commonly reported strategy followed by training, education and supervision. Both rules and training are behaviour based interventions that rely on individuals ‘doing the right thing’ to be effective. Use of training as a mitigation strategy to protect against risks associated with training presents an interesting dilemma for simulation. Boyle et al. [29] found that students’ perception of their performance using the defibrillator and what they actually do is *‘vastly different’*, and students in most cases failed to follow taught processes for defibrillation safety. Yet both Boyle [29] and Turban et al. [31] recommend rules and training as risk mitigation strategies for defibrillator risk, despite their own findings that these approaches are unreliable and may contribute to risk. Risk from sharps presents a similar dilemma [30]. These findings highlight an overreliance on strategies at the lower end of the HOC and a failure to consider more effective elimination, substitution or engineering strategies.

Instructional design plays a central role in anticipating and reducing foreseeable risks to SPs. Good instructional design is also a key strategy to combat negative learning [28]. The Association for Standardised Patient Educators (ASPE) standards of best practice [34] provide practical guidance to support good instructional design and recommends very deliberate strategies to protect SPs from harm. Collectively, these strategies reflect an understanding that participant safety must be embedded within both the physical and educational design of simulation activities. The HOC framework highlights that the importance of maximising elimination where possible, strengthening engineering controls to reduce dependence on lower-tier measures, and regularly reviewing administrative measures to ensure their continued effectiveness [22] .

### Need for governance

The literature highlights a clear need for robust governance of health and safety in healthcare simulation [4, 15, 30]. Although healthcare and education institutions typically have established health and safety governance structures, risk assessment processes, and safeguarding policies in place, these systems are not always effectively translated to or rigorously applied within simulation-based activities. Three key components of health and safety governance are commonly identified: simulation-specific risk assessment tools; simulation-specific adverse event reporting systems; and simulation safety policies with associated procedures. The literature emphasises the importance of collaboration in the design of governance systems. Collaboration should include all relevant stakeholders such as simulation designers, simulation faculty, technicians and organisational health and safety personnel [4, 5, 24].

In relation to risk assessment, simulation centres often rely on general organisational risk assessment policies [15]. However, such approaches may fail to capture the distinct and context-specific risks associated with healthcare simulation [24], reflecting a broader disconnect between existing organisational systems and their application to simulation practice. Lambert et al. [15] call for dedicated tools and processes that explicitly target simulation-specific risks, alongside greater consensus across the simulation community regarding standards for health and safety in simulation.

Similarly, in relation to adverse event reporting, the literature consistently supports the need for simulation-specific reporting systems. Traditional clinical incident reporting mechanisms do not adequately capture simulation-related hazards, resulting in underreporting and missed opportunities for organisational learning [4, 24, 27]. This further illustrates how existing organisational systems, while present, may be poorly aligned with the unique characteristics of simulation activities.

With regard to safety policy, guidance on the development of simulation-specific safety policies remains limited. Brazil et al. [4] provide one of the most comprehensive published approaches to safety policy development for a translational simulation programme; however, they acknowledge that the process is unclear and describe a “*piecemeal approach in most institutions*” (p. 2). Simulation societies can play a valuable role in bridging this gap by offering structured guidance and support for governance implementation [4]. For example, the Society for Simulation in Healthcare (SSH) provides a Simulation Centre Policy and Procedure Manual and Template [44], alongside accreditation standards that outline practical governance practices for effective health and safety management [45]. Collectively, these findings emphasise the importance of tailored governance structures for simulation, and of ensuring that existing organisational health and safety systems are actively understood, adapted, and consistently applied to support proactive risk management and a learning-oriented simulation culture.

### Recommendations for future research and practice

Relatively little is known about the nature and occurrence of adverse events in simulation. The extant literature focuses on a narrow range of rather predictable simulation risks. Risk from modern simulation technologies such as AR or VR, and risks associated with the use of biological materials are rarely mentioned in the published literature. Yet better insight into all forms of risk is required to inform effective prevention strategies. Future research should build on this initial work through broader data collection strategies such as surveys and qualitative interviews with diverse simulation roles and across a range of simulation settings. Such research would help to validate the risks identified in this study and elucidate how they arise. Additionally, further research is required to examine risk reduction strategies and to evaluate their effectiveness in mitigating risks associated with simulation activities. There is a need to develop practical tools to support simulation safety such as risk assessment templates, adverse event reporting templates and guidance to support the development of a simulation safety policy. These should be tailored specifically to simulation activities. This work should adopt a collaborative approach, engaging simulation practitioners, technicians, educators, and safety experts to ensure relevance, applicability, and impact. Future practice necessitates that simulationists develop awareness of existing health and safety governance and policy frameworks within their organisations, while also recognising the need for more simulation-specific tools.

### Limitations

There are several limitations to this scoping review. First, the grey literature was not included. Grey sources may have provided valuable insight into frontline practice, and context-specific challenges that may not appear in academic publications [46]. The approach taken here prioritised analytical rigour and conceptual clarity, with future empirical research well placed to extend these findings through practitioner-focused methods. A future review could focus specifically on simulation safety policies to identify examples of good practice that could be shared across the simulation community. Second, we chose not to include articles addressing COVID-19–related risks, as issues such as social distancing are no longer widely applicable. However, the ability of simulationists to recognise and respond appropriately to novel or emerging risks is important and might usefully be considered in other research. Third, the included studies were highly heterogeneous, meaning the synthesis relied heavily on reviewer interpretation. In this case, the analysis was informed by a multidisciplinary research team with expertise in human factors, safety science, healthcare simulation, evidence synthesis, and health and safety management, all with over ten years’ experience in healthcare simulation. As we note herein, this work will offer an important first step to subsequent work focused on developing tools and guidance to support health and safety risk management in simulation.

## Conclusions

Safety is fundamental to simulation yet simulation generates a unique set of risks that may be underappreciated. Risks to patients from in-situ simulation are well recognised and strategies exist to mitigate them. However risks to simulation participants are less predictable as they emerge from the complexity of the simulation environment and activity. In such circumstances robust risk assessment, risk reporting and safety policy are critical and must provide a foundation for higher- tier risk prevention strategies.

The findings from this review suggest that formal risk assessment is uncommon, safety policy is variable, safety reporting is limited, and there is an overreliance on lower-tier prevention strategies. This disparity underscores the need to develop simulation specific governance tools including standardised risk assessments, reporting systems for adverse events, and safety policies designed for simulation settings. These tools should be embedded within, and aligned to, the parent organisation’s health and safety governance process. Crucially, this work must be co-designed with those who design, deliver and support simulation, to ensure usability and relevance.

## Supplementary Information

Below is the link to the electronic supplementary material.

Supplementary Material 1.

Supplementary Material 2.

## Data Availability

All data generated or analysed during this study are included in this published article [and its supplementary information files].

## Abbreviations

AR: Augmented Reality
CDC: Centre for Disease Control
HOC: Hierarchy of Controls
SP: Simulated Participant/Patient
SSH: Society for Simulation in Healthcare
PPE: Personal Protective Equipment
VR: Virtual reality
