# Supplementary material for: Health and safety considerations for healthcare simulation: a scoping review of published literature
Source: Adv Simul (Lond). 2026 May 2;11:46. doi: 10.1186/s41077-026-00443-w (PMC13317352; doi:10.1186/s41077-026-00443-w)
Supplement: Supplementary file 1 — Supplementary Material 1. [file 41077_2026_443_MOESM1_ESM.docx]

Medline Search Strategy

SAFETY / RISK / HARM TERMS

1. (Health and Safety).ti,ab.
2. (Occupational Health and Safety) .ti,ab.
3. (Simulation adj1 risk*)
4. (Simulation adj1 safety)
5. (Simulation adj1 injur*)
6. (Simulation adj1 hazard*)
7. (Simulation adj1 harm*)
8. (Physical adj 1 risk*)
9. (Physical adj 1 harm*)
10. (Physical safety)
11. (Safety adj1 management)
12. (Risk adj1 prevent*)
13. (Risk adj1 mitigat*)
14. (Adverse event*)
15. exp Safety/
16. exp Risk/
17. exp Harm Reduction/
18. exp Safety Management/
19. exp Accident Prevention/
20. exp Risk Management/
21. exp Mitigation/
22. exp Patient Harm/
23. 1 OR 2 OR 3 OR 4 OR 5 OR 6 OR 7 OR 8 OR 9 OR 10 OR 11 OR 12 OR 13 OR 14 OR 15 OR 16 OR 17 OR 18 OR 19 OR 20 OR 21 OR 22

SIMULATION TERMS

1. Simulation.ti,ab.
2. (Simulation based education)
3. (Simulation event*)
4. (Simulation activit* )
5. (Simulation program*)
6. (Translational simulation).ti,ab.
7. (Simulation facilit*)
8. (Simulation center* or simulation centre*)
9. (In situ simulation or Insitu simulation)
10. (Clinical site* or Clinical setting*)
11. exp Simulation Training/
12. exp Patient Simulation/
13. exp Educational Facilities/
14. 24 OR 25 OR 26 OR 27 OR 28 OR 28 OR 29 OR 30 OR 31 OR 32 OR 33 OR 34 OR 35 OR 36

HEALTHCARE TERMS

1. Healthcare. MP.

Combined Search

1. 23 and 37 and 38
2. limit 39 to (English language and yr="2010 - 2025")
