# Supplementary material for: Health and safety considerations for healthcare simulation: a scoping review of published literature
Source: Adv Simul (Lond). 2026 May 2;11:46. doi: 10.1186/s41077-026-00443-w (PMC13317352; doi:10.1186/s41077-026-00443-w)
Supplement: Supplementary file 2 — Supplementary Material 2. [file 41077_2026_443_MOESM2_ESM.docx]

**Supplemental Data. Data Extraction Table.**

| **Author, title and date** | **Location** | **Publication type** | **Article focus and aims** | **Risks identified** | **Contributory**  **Factors proposed** | **Suggested mitigation strategies** | **Hierarchy of controls** |
| --- | --- | --- | --- | --- | --- | --- | --- |
| Bajaj, K., et al. (2018).  “No-go considerations” for in-situ simulation safety.” | USA | Commentary | In-situ simulation  The identification and establishment of ‘no-go consideration to determine when it is appropriate to launch or cancel an in situ simulation program | Risk to Patients from:   - the administration of simulated medications to a real patient - soiled equipment being returned to patient use - the use of actual resources (human and others) for simulation, when needed for patient care | Staff not recognizing the risks of in situ simulation | Collaboratively develop and agree ‘no go’ rules. Considerations are:   1. Staffing needs 2. Workflow patterns 3. Clinical load/Acuity 4. Equipment needs 5. Unanticipated events/Threats to psychological safety | Elimination |
| Boyle, M. J., et al. (2015).  "Assessing student paramedic visual and verbal checks for defibrillation safety—an observational study." | Australia | Research article | Location generic  Defibrillation safety  A prospective observational study to assess visual and verbal safety checks by paramedic students prior to defibrillation | Risks to Participants (learners and Staff) from:  The inadvertent “shocking” of the patient when another rescuer or bystander who has contact with the patient. | - Lack of training and experience using the equipment - Students failing to follow procedures | Study findings:   - Students’ perceived performance during the cardiac arrest simulation did not correlate with what the assessor observed during the simulation - Student perception of their performance and what they actually do is vastly different - Students in most cases failed to follow taught processes for defibrillation safety during the simulations   Prior to the defibrillator analysing the cardiac rhythm, to charging the defibrillator, and to “shocking” the patient the learner should:   1. verbalise “stand clear” 2. make eye contact with their partner   makes a visual scan of the immediate area  Mitigation strategies:   - Rules for defibrillator safety - Training in defibrillation before use in training | Administrative/  Training |
| Bradley, M. (2022).  “Content validity test of a safety checklist for simulated participants in simulation-based education in the United Kingdom: a methodological study.” | UK | Research Article | Location generic  To describe (i) the development of the SP safety checklist and (ii) the evaluation of feedback responses to assess whether SPs felt the checklist was safe | The risk of physical harm to SPs from:  Learners performing interventions on them | None identified | Good Instructional design supported by a SP safety checklist to be completed before and after the simulation event  Checklist contains 5 domains of items:   1. Prepara­tion 2. Pre-simulation pre-brief with SP 3. Delivery 4. Debrief 5. Evalu­ation   The first 2 domains are completed before the simu­lation event, and the final 3 domains afterward. Each statement is for discussion and action. Each statement was followed by a check box to ensure full coverage so the checklist should be used in its entirety | Elimination  Administrative |
| Brazil, V. et al. (2022).  “Developing a simulation safety policy for translational simulation programs in healthcare.” | Australia | Guidance article | Insitu simulation  To describe the process used to develop the simulation safety policy at a health Service, in order to provide practical guidance for practitioners working in translational simulation to manage health and safety | Risks to patients and health systems from:   - fake medications given to real patients - staff pre-occupied with treating a manikin when real patients require attention, - emergency call systems activated by mistake - simulated patients listed in a hospital’s electronic medical record - risk of spreading infection (such as COVID- 19) due to gathering staff together for training, - moving manikins between clinical spaces - using personal protective equipment that may be in short supply for real patients | The close physical proximity of simulated and real practice | A simulation safety policy  Recommendations for simulation safety policy development   1. Form a steering group and identify relevant stakeholders to contribute to policy development and approval 2. Identify existing safety procedures that are relevant for the simulation program 3. Incorporate simulation safety practices required in SSH accreditation processes *and* Raemer’s ‘Ten Commandments’ 4. Consider the nature and extent of predicted safety risks, based on reports in the literature and local experience—adverse events and near misses. Prioritize medication safety and liaise with health service pharmacy representatives 5. Effectively communicate the existence of the simulation safety policy, and the need for staff involvement and compliance 6. Enable simulation faculty to conduct safe simulation sessions that are compliant with the policy 7. Develop a reporting process for simulation related adverse events or near misses | Administrative |
| Hambridge et al (2022)    “An audit of sharps injuries in clinical skills  simulation wards at a UK University.” | UK | Original article - Audit | Academic setting.  An audit of all accidents on simulation wards in a nursing school | Risks to learners and staff from:   - Sharps, Fainting, Back injury, Facial injury, Slips, Splash to the eye | - Lack of experience - Underdeveloped skills - Lack of attention to personal safety | Study findings:  Personnel who sustained injury:   - Students 88% - Technicians 6% - Others 6%   This audit showed that 6.25% of sharps injuries involved a technician and 3.13% involved a cleaner  Mitigation strategies:   - Lecturers working within simulation settings adhere to the regulations regarding safe sharps usage - Providing opportunities for exposure to different devices through simulation - Use simulation to teach students the safe use and disposal of sharps - A thorough assessment of the various risks to health and safety in relation to the use of sharps - Better supervision during sharps training sessions this may aid the identification of unsafe practices which may contribute to injuries - Biosafety awareness training | Administrative/  Training |
| Hensel, D., et al. (2019).  "The physical demands and risks of working in healthcare simulation center." | USA | Research article  A questionnaire study of simulation operators and educators | Simulation Centre  To describe the physical demands and risks associated with working in a simulation centre | Risk to staff from:  Musculoskeletal disorders | Lack of systems to protect workers from harm | Study findings:  55% of participants believed that their job description did not match their work and 59% of participants believed that they had experienced an MSD because of their work in a simulation centre  Mitigation strategies   - Accurate job descriptions for simulation technicians - Written policies and procedures - Reporting systems - Engineering controls e.g. devices to lift heavy objects, work area design, equipment selection - Administrative controls e.g. rule and procedures to minimise exertion, duration, repetitive motions - Individual level strategies e.g. instruction | Administrative  Engineering |
| Lambert et al (2025)  “Risk assessment processes within healthcare simulation centers: A scoping review.” | Australia | Review article  . | Simulation centre  A scoping review to map the existing literature on risk assessment in healthcare simulation centres and identify how risk is assessed, measured and mitigated in these spaces | Risk to learners and staff from:  (i) manual handling, (including musculoskeletal injuries)  (ii) fainting and other medical episodes   1. needle stick injury (NSI)/sharps injury and injury from glass ampules. | Lack of a structured approach to risk assessment | There is a need for a more structured approach to risk assessment in simulation centre environments  Future research and development should focus on several key areas:   - The design and implementation of a standardised risk assessment model specifically for simulation centres to provide guidance for developing policies and procedures - The development of simulation centre-specific adverse event reporting avenues | Administrative |
| Lewis, K.L.et al. (2017).  “The association of standardized patient educators (ASPE) standards of best practice (SOBP)” | USA | Standards of SP practice | Location generic  To pronounce underlying values and to establish Standards of Best Practice (SOBP) that ensure the growth and integrity of SP-based endeavours | Risk to SP educators from:  Breaches of safety, confidentiality and respect | None identified | Each of the 5 domains of the SOBP speak to safety in an indirect way but (1.1) Safe Work Environment directly addresses safety concerns.  13 practice guidelines to protect SPs:  1. Ensure safe working conditions in the design of the activity  2. Anticipate and recognize potential occupational hazards  3. Screen SPs to ensure that they are appropriate for the role  4. Allow SPs to opt out of any given activity if they feel it is not appropriate for them to participate  5. Brief SPs so they are clear about the guidelines and parameters of a simulation activity  6. Provide SPs with strategies to mitigate potential adverse effects of role portrayal  7. Inform SPs and clients about the criteria and processes for terminating a simulation if they deem it harmful  8. Structure time and create a process for de-roling and/or debriefing  9. Monitor for and respond to SPs who have experienced adverse effects from participation in an activity  10. Provide a process for SPs and clients to report adverse effects  11. Support SPs who act in accordance with delineated program expectations if a complaint is made about them  12. Manage client expectations of an SP’s possibilities and limitations  13. Work with clients to clearly define the expected scope | Elimination  Administrative |
| Marshall, S., & McIntosh, C. (2017).  “Strategies for managing adverse events in healthcare simulations.” | Not specified | Book chapter | Location generic  To outline the preparation that should occur to prevent harm from simulation, and to discuss sources of physical and psychological injury and how they may be managed | Risks to patients and learners and staff from:  Adverse events in simulation:  Learning related adverse events e.g. **n**o learning occurs; Learning occurs but transfer fails; transfer occurs but the practice is applied inappropriately  Physical adverse events caused by :  Electrical devices, Clinical equipment, Defibrillators, Fluids, Gas supplies, Mannequins, Sharps injuries, Drugs and other clinical supplies, 3D and virtual reality technology, Real clinical events | - Poor simulation design - Poor planning and preparation for simulation | - Good instructional design to anticipate and avoid risk - The design of simulation facilities - Good health and safety processes - Preparation of the learners - Strategies to learn from adverse events.   The paper provides specific mitigation strategies for each identified risk:   - **Electrical devices:** A routine programme of basic electrical safety - **Defibrillators:** opt to have the defibrillators modified such that the electrical current is absent or minimal, or have the electrical energy ‘absorbed’ by a proprietary device before it reaches the mannequin - **Fluids:** Learners should be made aware of the risks of electrical shock and where possible separation of fluids and electrical equipment should be the norm. - **Gas supplies:** Compressed air can be used as a substitute for many gases, if some mannequins and devices require oxygen, Care must be taken to prevent sparks. Compressors used for the running of mannequins need to be bled after use and require regular servicing to ensure safe use - **Mannequins:** use *either* a live defibrillator *or* oxygen-enriched gases, but not both - **Sharps injuries:** Processes such as sharps disposal should be reinforced - **Drugs and other clinical supplies:** a clear policy on the use of real versus simulated drugs and clinical supplies - **Virtual reality:** appropriate simulator sickness minimization and response strategies - **Confederates and SPs:** Clear expectations should be communicated with participants - **Real clinical events:** clearly articulated local policy and procedure for dealing with medical emergencies, including how help is summoned and how to communicate ‘this is not a simulation!’ - **Dangerous clinical behaviour:** any dangerous behaviours observed in simulation must be addressed appropriately - **Adverse events:** Strategies should be in place to learn from adverse events related to simulation and to prevent future similar events | Elimination  Engineering  Administrative |
| Morse, C., et al. (2019).  “The changing landscape of simulation-based education." | USA. | Special feature | Location generic  Highlights the changing landscape of SBE and describes elements critical to its successful use | Risks to patients and learners from:   - The accidental activation of emergency responders to a simulation event - Disabled or expired equipment inadvertently used in a clinical setting - Witnessing a simulated emergency causing emotional responses for the learner by triggering past traumatic experiences - Simulated supplies entering the real health care supply chain | Lack of: planning, policies and learner education | - **Hospital-wide policies**: that address how supplies and equipment used in SBE are differentiated from authentic patient care items - **Prebriefing:** to raise awareness of risks, to remind learners which equipment is real and which is simulated, - **Rules:** prohibiting removal of any simulated medications from the simulation laboratory, and developing a checkout procedure to catch items forgotten in pockets - **Accidents and incidents**: should be reported and investigated in order for us to learn from them. Sharing these incidents with our colleagues is an important way to ensure safe simulation practices | Administrative |
| Petrosoniak, A., et al. (2017).  "In situ simulation in emergency medicine: moving beyond the simulation lab." | Canada and USA | Guidance article | Insitu simulation  To present the uses of insitu simulation in Emergency Medicine and to present, implementation and mitigation strategies for ED specific challenges. | Risks to patients from:   - Potential interference with concurrent patient care - Cross contamination between simulation and clinical site of Equipment and /or medications | - Poorly timed simulations - Equipment and roles different from the normal workplace | The benefits of ISS should outweigh any potential harms.   - Plan sessions during historically low-volume times in the ED, to reduce participant stress - Establish a priori cancellation criteria - Decide in advance what equipment/medication will be taken from the ED and what can be brought in from non-operational supplies - Establish a full restocking process immediately following each scenario - Label all equipment and medication brought into the clinical environment as ‘simulation-only’ and use a checklist to ensure removal of such equipment | Eliminating  Administrative |
| Raemer, D. B. (2014).  "Ignaz semmelweis redux?" | USA | Commentary | Insitu simulation  A lesson to beware of unintended consequences, especially with in situ simulation | Risks to patients from:   - Simulated medications being given to real patient - Simulated equipment or supplies getting into real clinical environment - Real equipment returned unsterile or not ready for use after simulation - Real resources used for simulation - Inadvertent negative teaching | Under appreciation of risks by staff, faculty and learners | - Simulation only labelling of medications, using real medications, medication accounting - Simulation only labelling of equipment and supplies, secure locking and strict clean up procedures - Keeping everything for simulation locked up and inaccessible - Educating staff and instructors on the hazards of simulation and creating a culture of safety within the simulation program - Treat certain simulations, such as unannounced mock events, as if they were real by using only real medications and supplies - Informing participants about the hazards of simulation for each and every event - Informing patients and families about planned simulation activities - Contingency planning (.i.e. develop ‘no go’ rules) - Pre-meetings with everyone in the training environment - Policies and procedures to achieve and maintain safety in simulation. - Getting everyone involved in simulation to be knowledgeable about policies and procedures and adhering to them. - Policies should keep pace with changes in practice | Elimination  Administrative |
| Raemer, D., Hannenberg, A., & Mullen, A. (2018).  “Simulation safety first: an imperative.” | USA | Editorial | Insitu simulation  To highlight the possibility of simulation-related mishaps resulting in patient, participant, staff, or bystander harm, and to present the merits of taking a systematic approach to identifying and mitigating this safety risk of simulation | Risks to patients, and learners from:   - Medications and supplies become interchanged - Nursing or medical student practicing injections on each other using unsterile educational supplies - Devices modified in some way for simulation and real equipment being exchanged - Defibrillator cables designed for use on mannequins being put onto a crash cart intended for patient use - Defibrillators being used for simulation could leave clinicians without it when needed for a cardiac arrest - Institutional systems such as patient records can be misused during simulations - Resources such as resuscitation teams, blood bank, first responders, etc being mistakenly called during a simulation exercise - Staff not responding to an emergency call mistakenly believing it is for a simulation - Negative learning from shortcuts taken to implement the simulation efficiently might mislead a learner | Modification made to suit simulation | - Initiative a failure mode effects analysis (FMEA) for each simulation program - Adopt the Foundation for Healthcare Simulation Safety (FHSS), 10-item “pledge” of “best practices” also known as the 10 commandments of healthcare simulation safety - Prominently labelling medication, supplies, and equipment to indicate whether they are not for human use or not - Controlling access to simulation supplies, equipment, and spaces. - Using real medications and supplies when simulation is conducted in or near a clinical environment - Before and after session accounting of medications and supplies - Strict institution-wide policies and procedures to address specific hazards such as protocols for conducting and cancelling in situ simulations (i.e develop ‘no-go’ rules) - Communicating the risks and policies for safe simulation practice to staff, participants, and others | Elimination  Administrative |
| Riem, N., et al. (2011).  "Setting standards for simulation in anaesthesia: the role of safety criteria in accreditation standards." | Canada | Special article | Location generic  To describe a critical event which occurred in a simulation centre, and to review possible safety issues for participants and staff involved in medical simulation training  To raise the question of safety training in simulation centres | Risks to learners and staff from:   - using real anaesthesia machines, ventilators, or surgical tools such as scalpels, forceps, and surgical cautery - use of anaesthetic gases and nitrous oxide - Sharp instruments and needles and real drug vials - Electrical accidents, in general, and accidents due to live defibrillation - Risk of fires and explosions in a mock OR - Trip-and-fall injuries | - The drive for realism in simulation resulting in the accumulation of various real devices acquired from the workplace and used in full-scale or immersive simulation scenarios - Inexperienced trainees | - Analysis of training accidents and create an incident reporting system - Identify possible risks or hazards of used equipment and implement a schedule for periodic maintenance checks by a technician - Equipment must meet official safety and quality standards, and electrical devices go through regular inspections - Regulation of storage, use, and disposal of syringes, needles, and drug vials - Strategies to ensure personal safety, e.g., use of gloves and antistatic shoes - The education community and medical institution should be responsible for the establishment of a safety plan - Strive for consensus and compliance amongst the simulation community to a framework of standards for simulation based education, research and assessment - The institution responsible for the simulation centre should ensure that existing safety regulations are followed as recommended | Engineering  PPE  Administrative |
| Torrie, J., et al. (2016).  “Fake and expired medications in simulation-based education: an underappreciated risk to patient safety.” | New Zeland | Viewpoint | Location generic  To consider risks associated with the use of medications in simulation and discuss strategies to reduce risk | Risk to patients from:   - Contamination of the clinical environment with fake or expired medications intended for simulation use only.   Contamination can occur in several ways:   - Via clinical procurement and distribution routes, - By inadvertent carriage by personnel returning from a simulation area, - When fake or expired medications are unintentionally left in a clinical area after in situ simulation. - Differences in appearance between real and fake medications are often subtle. - Misidentification errors due to unclear labelling. | None identified | STRATEGIES TO REDUCE CONTAMINATION   - Use only real, ‘in-date’ medications.. - Manage all medications, fake or real, as required for real medications, engaging pharmacy staff to ensure that procedures align with clinical practice - Separate fake medications from the clinical supply chain - Separate the simulation supply chain from the clinical supply chain - Require clinical institutions to have separate systems for the purchasing, handling and storage of fake medicines, with tracking of supplies and disposal - Use labelling to alert users to the nature of fake medications - Alter the labels to alert clinical users to their nature - Develop systems for pre-reconciliation and post-reconciliation of fake or expired medications - Limit inadvertent carriage of fake or expired medication by requiring learners and staff to empty their pockets on departure - If fake or expired medications must be used, security measures should be in place for supply, storage, dispensing and audit - The considerations for management of   fake and expired medication also pertain to medical devices adapted or recreated for educational purposes | Elimination  Administrative |
| Turban, J. W., et al. (2010).  "Live defibrillation in simulation-based medical education--a survey of simulation centre practices and attitudes.” | Hawaii | Empirical investigation article | Simulation Centre  To present a survey study that was designed to assess:  a. the prevalence of live defibrillation use during training scenarios in healthcare simulation  b. Simulation centres’ policies and practices around live defibrillation  C. Whether trainees can use defibrillation equipment safely | The risks to learners and staff form:  Poor defibrillation technique | The drive towards high fidelity simulation | Study findings  There was incongruity between attitudes and practices, regarding the use of defibrillator safety training in simulation  The majority (89%) of responders considered live defibrillation an important element of simulation-based education and that 87% felt there was a need for formal defibrillator training before using live defibrillation, however, just over a third (36%) of the centres in this survey had a formal training policy in effect.   - Simulation centre management should assure adequate defibrillator safety training for all students, faculty, staff, and other users before actual defibrillator use during simulation based education and training - Simulation centre administrators should consider implementing a safety policy, assuring user demonstration of minimum competency before use of live defibrillation during simulation training | Administrative |
